# Tongue-in-Groove: A Novel Implant Design for a Blow-Out Fracture

**DOI:** 10.3390/jcm13061766

**Published:** 2024-03-19

**Authors:** Je-Yeon Byeon, Yong-Seon Hwang, Hwan-Jun Choi, Da-Woon Lee, Jun-Hyuk Kim

**Affiliations:** Department of Plastic and Reconstructive Surgery, Soonchunhyang University Hospital Cheonan, Cheonan 31151, Republic of Korea; wpdusqus@gmail.com (J.-Y.B.); 135421@schmc.ac.kr (Y.-S.H.); 71830@schmc.ac.kr (H.-J.C.); 103022@schmc.ac.kr (D.-W.L.)

**Keywords:** orbital implant, surgical fixation devices, orbital fracture, operative, surgical procedure

## Abstract

**Background**: During blow-out fracture surgery, restoration of the orbital volume and rigid implant fixation are essential. The migration of an implant is a concern of most surgeons. The purpose of this study was to introduce a simple idea of molding and fixing an orbital implant. **Methods**: In the tongue-in-groove method, an incision of about 2 mm was made on the edge of the implant and it was bent to form a slot. A hole was made in the center of the implant for fitting a bone hook, and the implant was firmly fit into the remaining intact bone. Before and after surgery, computed tomography (CT) was used to evaluate changes in the orbital volume and the location of the implant. Statistically significant restoration of the orbital volume was confirmed on postoperative CT. **Results**: Compared with the unaffected orbital volume, the affected orbital volume was increased from 87.06 ± 7.92% before surgery to 96.14 ± 6.11% after surgery (*p* < 0.001). There was one case of implant migration during follow-up. However, the degree of movement was not severe, and there were no events during the follow-up period. **Conclusions**: The tongue-in-groove technique offers advantages, such as easy fixation of the implant, with minimal trauma to the surrounding tissues. In addition, the method offers advantages, such as being easy to learn, requiring little time for trimming the implant, and being relatively low cost. Therefore, it can be one of the options for implant fixation.

## 1. Introduction

Precise restoration of orbital continuity and rigid orbital implant fixation are the most important goals of orbital fracture surgery. Orbital fracture surgery has been studied in various aspects, including incision position, approach method, soft tissue restoration method, implant fixation method, and implant shape and mechanical properties [1,2,3]. For example, a patient-specific implant can be used through a mirroring technique that reconstructs the orbit on the affected side based on the orbit on the opposite normal side. This patient-specific implant allows for precise orbital wall reconstruction, which is closely related to good surgical outcomes [4,5,6,7]. A new approach to the fracture site using an endoscope, which can repair the herniated soft tissue and the fracture site, is also being studied [2]. Recently, it has become possible to check the implant location or bone graft in real time using a navigation tool or cone beam computed tomography CT [8,9].

However, even if precise orbital continuity is restored, unsatisfactory surgical results may result if the implant migrates, and unexpected side effects may also occur [10,11,12]. In addition, it is important to minimize surgical trauma to the eyeball and surrounding tissues, especially to the neurovascular bundle during the surgical procedure. In particular, damage to the blood vessels that penetrate through the bones can cause bleeding [13,14]. Excessive pressure to the eyeball during surgery can result in various side effects, such as oculocardiac reflex [15]. Tissue injury caused by excessive traction on the surrounding skin and mucosa can lead to an inappropriate surgical scar or eyelid malposition [16,17,18]. Therefore, an ideal surgical procedure should not only aim to fix the implant at the fracture site with minimal manipulation but also minimize the risk of implant migration. The purpose of this study was to introduce a simple idea of molding and fixing an orbital implant using the tongue-in-groove technique.

## 2. Materials and Methods

This study was a retrospective cohort study that involved patients diagnosed with a blow-out fracture (BOF) who were admitted to the Soonchunhyang University Cheonan Hospital from December 2017 to June 2023. A total of 339 patients were diagnosed with an orbital fracture and underwent surgery with an implant. During the study period, three plastic surgeons performed the surgery. Their medical records and computed tomography (CT) findings were reviewed retrospectively. The study protocol conformed to the ethical guidelines of the Declaration of Helsinki. It was approved by the Soonchunhyang University Hospital (Cheonan, Republic of Korea) human research review committee and the Institutional Review Board (IRB) of Soonchunhyang University Cheonan Hospital (IRB FILE No.: 2023-04-053-006). All participants provided written informed consent for the publication before study.

### 2.1. Patient Selection

Study inclusion criteria were as follows: (1) age > 15 years; (2) diagnosis of an orbital fracture; (3) indications for orbital fracture surgery (A. double vision caused by incarceration of muscle or the fine ligament system, documented by forced duction test and suggested by CT scans, B. radiographic evidence of extensive fracture, such that enophthalmos wound occurs, and C. enophthalmos or exophthalmos produced by an orbital volume change.); (4) surgery performed within 3 weeks of onset; (5) tongue-in-groove technique implant surgery; (6) normal vision; and (7) follow-up for more than 3 months. Study exclusion criteria were as follows: (1) damage to the eye and optic nerve, (2) missing preoperative and postoperative medical records, (3) missing facial three-dimensional (3D) CT before and after surgery, (4) bilateral orbital wall fractures, (5) orbital tumor, (6) thyroid-associated ophthalmopathy, (7) inferomedial orbital strut fracture, and (8) in combination with a zygomaticomaxillary complex fracture or a naso-orbito-ethmoid complex fracture.

Based on the above criteria, 163 patients who did not meet the inclusion criteria were excluded from the 339 subjects. As a result, 176 patients were enrolled in this study, in whom the tongue-in-groove technique was used. During the follow-up process, 38 patients were lost to follow-up. Finally, 138 patients were analyzed. The primary aim of the study was to evaluate the efficacy and safety of the orbital reconstruction tongue-in-groove implant technique. This was performed by first calculating the differences in the orbital volume between the reconstructed and normal sides and then determining the variances of the differences in the orbital volume. In addition, postoperative adverse events, such as implant migration, diplopia, enophthalmos, exophthalmos, hypoesthesia, retrobulbar hematoma, and decreased visual acuity, were evaluated. Preoperative and postoperative ophthalmological examinations (including diplopia, exophthalmometry, and EOM range) were performed in the ophthalmology department. Follow-up was performed in plastic surgery and ophthalmology outpatient departments.

### 2.2. Surgical Procedure

**Figure 1:**
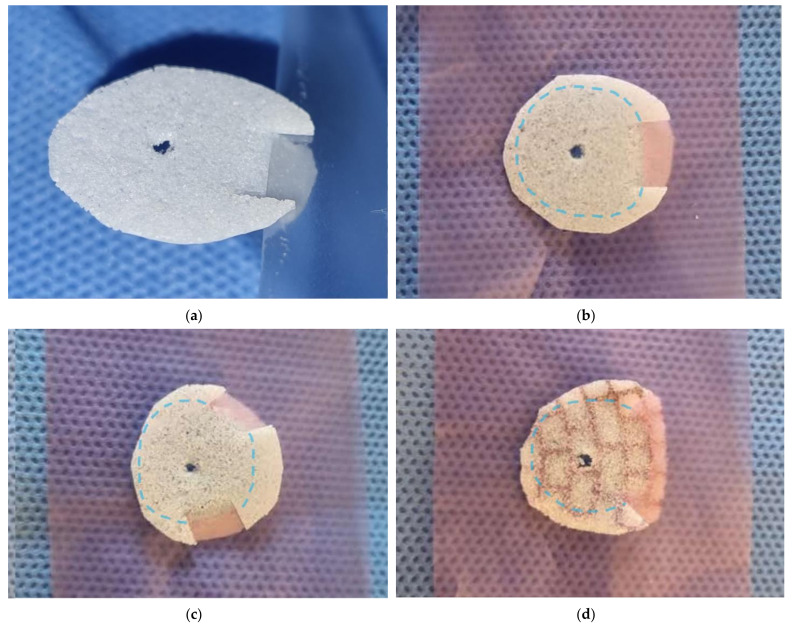
Making a tongue-in-groove at implant involved the following. An incision of at least 2 mm is made in the margin of the implant. Then, bend between the incisions to make a groove. In consideration of the shape of the fracture and the relationship of the surrounding intact bone, the gap or number of incisions can be adjusted. In the center of the implant, a hole large enough for a bone hook to adjust position of the implant is made. (**a**) The photo shows how the implant is fixed as tongue-in-groove. (**b**–**d**) The implant was fixed in tongue-in-groove technique on the fracture site marked with a blue dotted line. In this way, the type and size of the implant can be determined according to the shape and size of the fracture, and the position and spacing of the incision can be adjusted.

The tongue-in-groove implant technique involved the following. The implant was designed so that it was suitable for the defect size and shape based on the preoperative facial 3D CT and intraoperative findings, but the size of the implant was similar to the fracture site, or it was 2~3 mm larger to cover the fracture site. Using a scissor or No 11. blade, incisions at least 2 mm long were made on the edge of the implant. Then, the edge of the implant was bent between the incisions so that they formed a lowercase y shape when viewed from the side (Figure 1). The most important thing was to insert this bent groove into the intact bone at the fracture site. Therefore, it was necessary to adjust the part of the implant to be grooved, how spaced the grooves were, or how many grooves were required to be made according to the fracture pattern.

More grooves provide a more secure fixation but make it difficult to fit in precisely. Therefore, an appropriate number of grooves should be used according to the fracture pattern. In most cases, the groove was molded so that it could fit in front of the fracture site. However, concerns that the implant will move towards the orbital apex can be addressed by creating an additional groove at the posterior side of the implant. If the front groove and the back groove are positioned at a 180-degree angle, it is very difficult to insert the implant into the defect. Positioning the angle between the two grooves at up to 150 degrees, as shown in Figure 1c, is a way to easily insert the implant into the defect and hold it in place. In addition, in a medial blow-out fracture, if the fracture site is upwards, the implant may occasionally move in a forward upward direction. To prevent this, the groove can be fixed to the front and top with a wider width of the groove, as in Figure 1d, so that it can be fixed stably and easily, even in narrow surgical fields.

All patients in this study underwent surgical reconstruction of the orbit with either a Medpor implant (Stryker, Kalamazoo, MI, USA) and a Synpor implant (DePuy Synthes, West Chester, PA, USA). Porous polyethylene implant was mainly used; however, porous polyethylene implant containing titanium mesh was also used.

**Figure 2:**
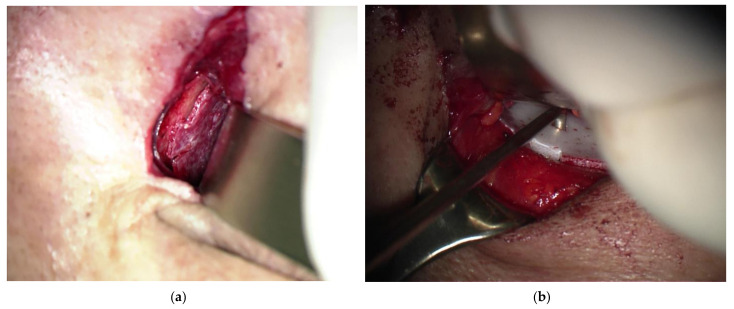
Intraoperative manipulation of implant. (**a**) Intraoperative picture of a patient with a medial orbital wall fracture. Place the implant over the fracture site. Next, adjust the position so that the groove of the implant fits into the front intact bone. The bone hook is convenient for adjusting the position of the implant in a small space. (**b**) Intraoperative picture of a patient with an inferior orbital wall fracture. It shows that the groove of the implant is inserted into the intact bone by inserting the bone hook into the hole in the center of the implant and then pulling the front.

**Figure 3:**
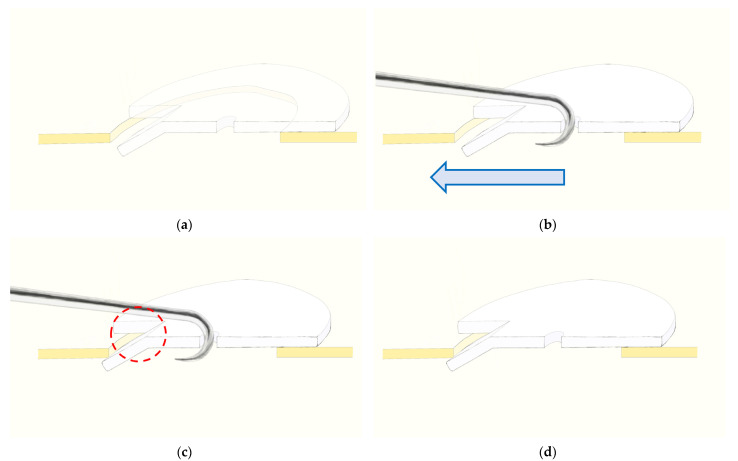
Schematic diagram of the surgical procedure. (**a**) The implant is placed above the fracture site. At this time, it is placed slightly back from the fracture site. (**b**) The bone hook is threaded into the center hole of the implant and force applied in the direction of the blue arrow. (**c**) The implant’s groove is adjusted to fit into the intact bone (red dotted line) (**d**) The bone hook is removed and the stability of implant is checked. In most cases, the position of the implant was determined after a forced duction examination. As needed, the navigation tool was used to check the change in the position of the implant.

**Figure 4:**
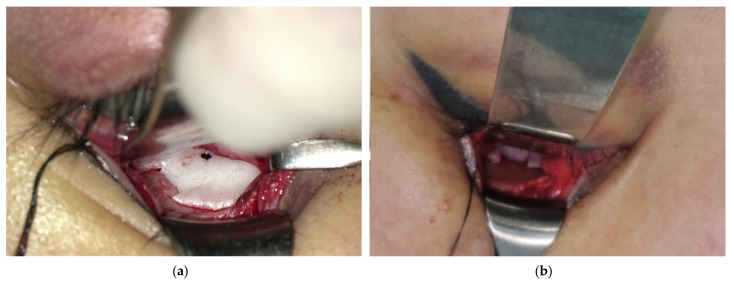
Intraoperative findings: (**a**) Intraoperative photograph of a patient who had a fracture in the anterior portion of the inferior wall. It can be seen that the implant is fixed to the intact bone. (**b**) Intraoperative photograph of a patient who had a fracture in the posterior portion of the inferior wall.

**Figure 5:**
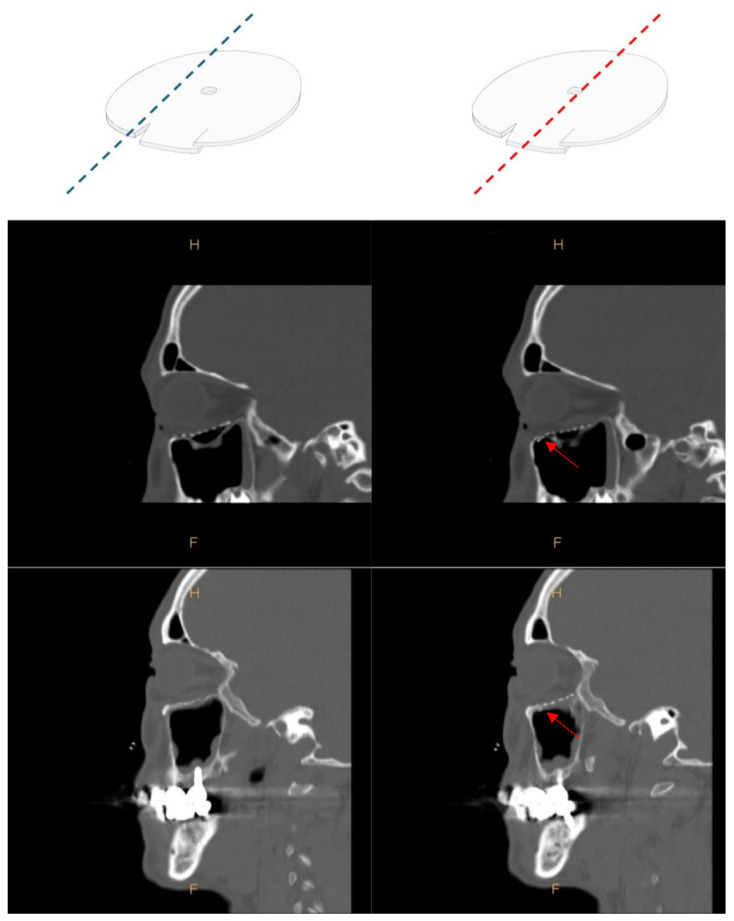
Tongue-in-groove implants as shown on CT. For intuitive understanding, we have attached the CT findings of the implant containing the titanium mesh. The pictures on the (**left**) are the CT results corresponding to the blue dotted line. It is confirmed that the implant is located above the intact bone. The pictures on the (**right**) are the CT results corresponding to the dotted red line. The groove in front of the implant (red arrow) is tucked into the underside of the intact bone.

The operation was performed under general anesthesia. Fractures of the inferior orbital floor were approached using a transconjunctival approach or a subciliary incision, and fractures of the medial orbital walls were approached using a Lynch approach or a transcaruncular approach. Meticulous dissection was performed using microscopy or a Loupe magnifying glass to identify the fracture sites, and implants were inserted precisely. The implant was inserted along the intact bone around the bony defect to the posterior side of the fracture site so that the groove of the implant was located in the defect. Then, a bone hook was inserted into the hole of the implant, and it was pulled out to fix the groove of the implant by inserting it into the intact bone (Figure 2 and Figure 3). After the forced duction test of the eyeball, it was confirmed that the implant remained rigid (Figure 4 and Figure 5).

**Figure 6:**
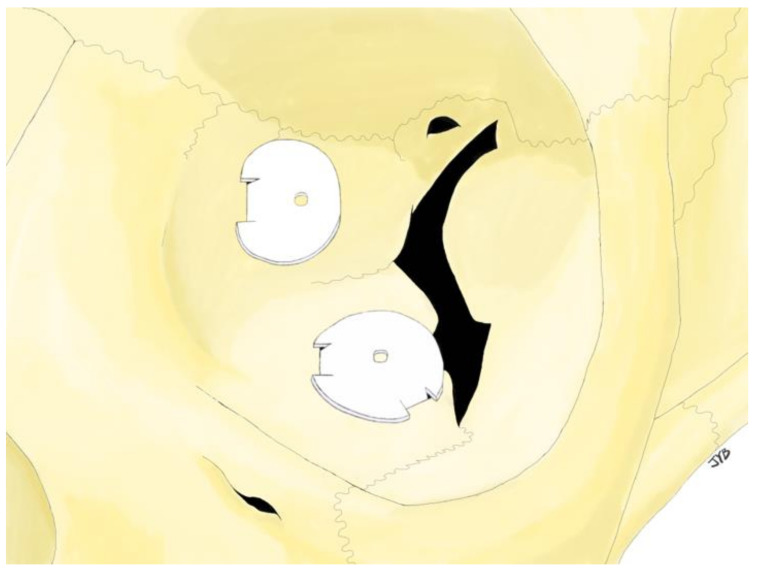
Schematic diagram of implant insertion: Depending on the shape and size of the fracture, the number, size, and spacing of grooves can be adjusted. The more grooves the implant has, the stronger the fixation, but the more difficult it is to fit it into the fracture site. The groove of the implant is located in front of the fracture site for ease of insertion and prevents migration to the front. Even a single groove, if used properly, can achieve sufficient fixation (medial orbital wall). However, if it is difficult to obtain enough hold from one groove, it is better to use two or more grooves (inferior orbital wall). Therefore, it is important to determine the spacing, number, and location of the groove depending on the fracture pattern.

This surgical method can be used for both inferior wall fractures and medial wall fractures. In particular, it can be useful for medial wall surgery with a narrow field of view compared to inferior wall surgery with a wide field of view (Figure 6). The periosteum was closed with 6-0 Maxon. In cases of a subciliary approach and Lynch approach, the skin incision was closed with 7-0 Nylon. In cases of a transconjunctival approach and a transcaruncular approach, the conjunctiva was closed with 8-0 Vicryl. Supplemental Digital Content demonstrates the insertion and position of implant. One groove was used for fixation. After placing the implant above the defect area, the position of the implant is adjusted using a bone hook device so that the groove is inserted between the intact bones.

### 2.3. Orbital Volume Measurement and Exophthalmometry Measurements

**Figure 7:**
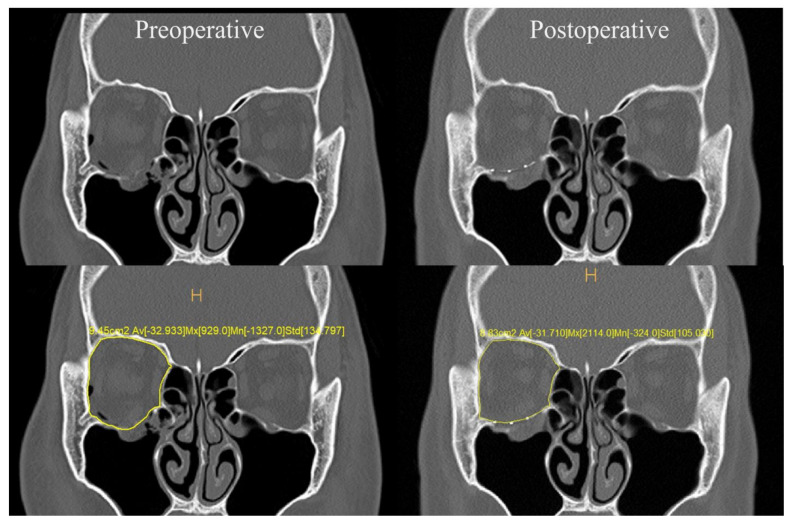
Preoperative and postoperative CT. Soft tissue herniation is observed in right inferior orbital wall fracture. Synpor^®^ porous polyethylene implant containing titanium was used because of the wide fracture area. The implant was fixed using a tongue-in-groove technique. Postoperative CT shows a well-positioned implant compared to the unaffected side. Deja-view software was used to calculate the volume of both orbits (yellow).

Facial 3D CT scans were obtained before surgery and on the third day after surgery. On preoperative facial 3D CT, the orbital volume of the normal and affected sides was measured. Subsequently, the changed orbital volume was measured on the postoperative facial 3D CT to compare the degree of improvement. On CT scans, the polyethylene implant can be seen as a low-hounsfield unit that is distinct from normal tissue when enlarged in detail. For implants that contain titanium, the implant is easy to identify. Therefore, the volume of the orbit was accurately measured. The degree of change in orbital volume was calculated as the sum of the areas through the area measurement tool in the Deja-View software, (https://www.dongeunit.com/deit/index.do, accessed on 18 March 2024) (Dongeun it, Seoul, Republic of Korea) (Figure 7). The extent of the difference was analyzed by comparing the preoperative volume and the postoperative volume with the normal volume.

Also, the effect of orbital wall reconstruction was quantified using the orbital volume reconstruction rate (OVR%), which was calculated as follows: OVR = (1 − (A − B)/B) × 100 (%); A, volume of the fractured orbit after surgery; B, volume of the contralateral orbit. Thus, when the fractured orbit volume after surgery equals that of the contralateral orbit, OVR% is 100% [19]. Descriptive statistics were used to evaluate the baseline characteristics, with mean and SDs used for quantitative variables and counts and percentages for categorical variables. Continuous parametric data were analyzed by the paired sample *t* test. A *p* value of less than 0.05 was considered statistically significant, and all analyses were conducted using Rex Pro software version 3.6.3 (Rex soft, Seoul, Republic of Korea).

## 3. Results

**Figure 8:**
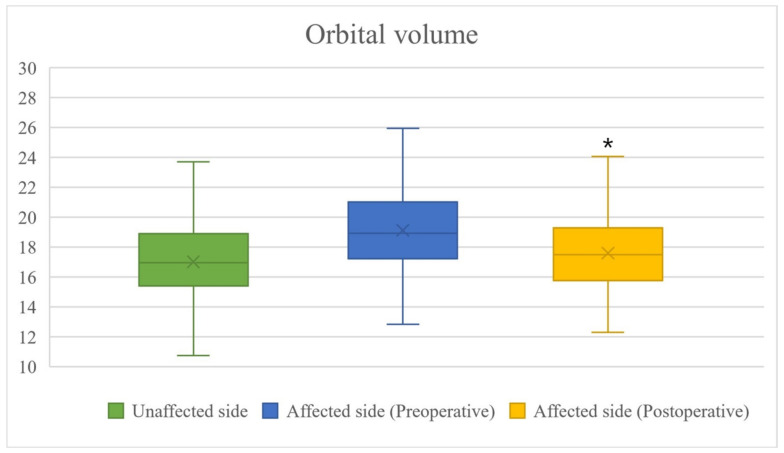
Difference of orbital volume before and after surgery: The normal orbital volume was found to be 17,006.16 ± 2444.30 mm^3^, the preoperative orbital volume was 19,113.51 ± 2530.86 mm^3^, and the postoperative orbital volume was 17,589.43 ± 2325.34 mm^3^. At this time, it can be confirmed that the volume of the orbit after surgery is statistically significantly similar to the volume of the normal orbit. However, compared to normal orbital volume, the postoperative orbital volume seems to be a little larger. Occasionally, it was overcorrection, but overall, it was undercorrection. * *p* < 0.0001.

Among the total 138 patients, 55 had fractures in the medial wall, 55 in the inferior wall, and 28 had fractures in the medial and inferior walls (Table 1). The orbital volume of the unaffected side was measured as 17,006.16 ± 2444.30 mm^3^, the preoperative orbital volume of the affected side was measured as 19,113.51 ± 2530.86 mm^3^, and the postoperative orbital volume of the affected side was measured as 17,589.43 ± 2325.34 mm^3^ (Figure 8).

On comparing the orbital volume of the unaffected side and that of the affected side before surgery, there was a difference of 2107.35 ± 1216.62 mm^3^, which was not statistically significant (*p* = 0.1129). On the other hand, on comparing the orbital volumes of the unaffected side and the affected side, there was a difference of 583.27 ± 891.21 mm^3^, which was statistically significant (*p* < 0.0001). Similarly, the preoperative OVR was 87.15 ± 7.75, which was a statistically insignificant result (*p* = 0.4421), and the postoperative OVR was 94.21 ± 6.06, which was a statistically significant (*p* < 0.0001) improvement in the orbital volume (Table 2).

Diplopia occurred in 42 patients before surgery. Hypoesthesia occurred in 19 patients, and enophthalmos occurred in 12 patients. One patient had a retrobulbar hematoma at the time of injury, but it improved after appropriate treatment and underwent subsequent surgery. In terms of postoperative complications, diplopia occurred in 22 patients, hypoesthesia in 15 patients, enophthalmos in six patients, ectropion in one patient, and implant migration in one patient. In most of the cases, diplopia improved within 3 months. However, diplopia persisted in one patient even after 3 months; thus, revision surgery was performed after 6 months. Enophthalmos occurred in four patients who underwent simultaneous inferior and medial wall surgery and in one patient who underwent medial wall surgery and one patient who underwent inferior wall surgery. After the subciliary approach, ectropion developed in one patient, but symptoms improved after 6 months. Implant migration was confirmed on postoperative CT in one patient, but the degree of displacement was not severe and there were no clinical symptoms; thus, follow-up was performed. No complications, such as retrobulbar hematoma or blindness, occurred (Table 3).

## 4. Discussion

The migration of implants after BOF surgery is a concern for many surgeons. In Seifert’s study, 34 (2.1%) of the 1594 patients with an orbital fracture showed displacement of the implant [20]. In addition, in Ho’s study, one of the 26 patients with an orbital fracture experienced a palpable anterior rim of the implant due to early migration of the implant [21]. Dancey reported on the late migration of implants that occurred 25 years after BOF surgery [11]. Likewise, Weintraub reported late implant migration that occurred 15 years after BOF surgery [12]. As such, if the implant is not properly fixed after BOF surgery, both early migration and late migration may occur, resulting in various side effects and complications [22,23]. If the implant migrates backward, it can cause damage to the optic nerve and muscles that move the eye [24,25]. On the other hand, if the implant migrates forward, it can cause irritation of the skin and soft tissues and inflamed tissue, and in severe cases, it can cause infection or skin injury [10,11]. Most implant migration occurred in the anterior direction. Therefore, the author mainly chose the method of locating the groove of the implant’s anterior side. However, depending on the pattern of the fracture, it is recommended to adjust the position and number of grooves.

Various surgical methods are being studied to prevent the migration of these implants. One of them is the fixation method, which involves physically inserting the implant into the defect. Kim introduced the inlay implanting method with multiple layers of the implant placed into the defect in medial BOF surgery. However, the disadvantage of this method is that there is an excessive amount of implant and space between implants, which can lead to the risk of infection [26]. Alternatively, the implant is fixed by inserting it in a Gamma shape, which has a disadvantage as the thread that holds the two implants may loosen, and a relatively thick onlay implant [27]. There is also a method of attaching a bone fragment to the implant and inserting it in the onlay method, which has an advantage as it can cover the defects while maintaining the bony continuity well. However, there is also a disadvantage that the thread can be loosened, and there may be a risk of migration because the implant is not firmly fixed [28].

Another fixation method is to fix the implant with screws, which can be further divided into two main types: fixing the implant directly on the inferior orbital floor [13,29] and fixing the implant on the inferior orbital rim [4,7,30]. In order to fix the implant directly using screws on the orbital floor, an angled screwdriver is required to fix the screws vertically. Also, screws are difficult to use, especially when the posterior wall is fractured. The limitation is that the screw must be driven at an angle rather than at a right angle [29]. In addition, in the process of drilling the screw, there is a possibility of damage to the blood vessels, nerves, surrounding tissues, and sinus mucosa around the orbital wall [13]. In addition, since the ethmoid sinus and maxillary sinus wall can be a source of infection, it is important to minimize the damage with minimal manipulation of the sinus [31]. Another fixation method is to extend the implant to the rim and fix it to the rim side instead of the orbital wall [6,32], which has a disadvantage as it can cause an excessively large implant compared to the fracture site and it may not be in perfect contact with the orbital wall [7,30]. In addition, for rigid fixation of the implant, at least two screws must be inserted [32]. The fixation method using screws has a limitation as the screw may break during the fixation process, or the screw may loosen after fixation [33].

In recent years, patient-specific implants have shown good surgical results [20]. Most customized implants are based on the shape of the orbital floor on the normal side and the mirroring technology is used to reconstruct the orbital floor on the fracture side [34]. However, sometimes the orbital volume is undercorrected or overcorrected after surgery, which may be due to the fact that the patient customizing plate is not corrected as planned, or because the shape of the orbits on both sides is different [5,35]. Fundamentally, a question also arises as to whether the two orbital floors look exactly the same in the mirror image. Andrades’ study found that in a study of 199 normal subjects, the volume of both eyes was not exactly the same [36]. In a study of Hertel exophthalmometry in healthy subjects, 71.6% of the patients had no significant difference in the exophthalmometry results in both eyes, while 18.4% of the patients had a difference of more than 1 mm in the exophthalmometry results in both eyes [37]. Therefore, in our study, a similar principle could be used to explain why the postoperative orbital volume and the normal orbital volume were overcorrected or undercorrected. Also, there are two other possible reasons for this. The first reason is that there are irregular areas in the orbit [38]. However, implants do not reflect these anatomical features, so they tend to be flat or concave. Therefore, the undercorrection of the orbital volume occurs due to the failure to properly correct those irregular areas. The second reason is when the fracture site is too close to the orbital apex. In this case, the author did not insert the implant too deeply. This is because it may cause traumatic optic neuropathy or be difficult to control bleeding. Therefore, this may be the cause of an increase in orbital volume after surgery and may lead to the development or worsening of enophthalmos in some patients.

Zimmerer’s study of 195 patients reported that fractures occurred most often in the middle third, followed by the posterior third and the anterior third of the floor [5]. Also, in this study, fractures of the medial orbital wall were found to be the same frequency as fractures of the inferior orbital wall, which was similar to Choi’s study [39]. Compared with inferior orbital wall fractures, medial orbital wall fractures have a narrower surgical field of view and are more difficult to operate. Therefore, there is a need for a surgical method that can effectively fix the implant in deep and narrow spaces. The author usually uses one tongue-in-groove pattern, but if the fixation is not enough, it can be changed to multiple tongue-in-groove patterns. Alternatively, it can be transformed by making a groove inside the implant and inserting it. Optionally, two holes can be made for the bone hook. This hold is useful for adjusting the position when inserting the implant, especially at a deep portion, can be expected to play a role in lowering the retrobulbar hematoma rate [40]. In addition, it has an advantage as it is convenient when removing the implant compared to the fixation method using screws [41]. Just apply force in the opposite direction of the groove and then remove the implant.

Common postoperative side effects may include diplopia, enophthalmos, infection, and eyelid malposition [18]. Acute side effects may include intraoperative bleeding and retrobulbar hemorrhage due to excessive dissection and deep implant insertion. Additional late side effects may include infraorbital nerve dysfunction, implant migration, postoperative optic neuropathy, blindness, and late retrobulbar hematoma [42,43]. The ocular complication rates range from 2.7% to 90.6%, depending on the inclusion criteria. Among them, severe ocular complications can occur in 10% to 13.7% of cases [44,45,46,47]. According to Seifert’s study, 12.9% of the patients experienced hypesthesia, 4.1% experienced diplopia, 4.0% experienced intra-orbital hematoma, 2.1% experienced implant displacement, 1.2% experienced exophthalmos, and 0.6% experienced visual impairment [20]. In this study, the rate of implant migration was 0.7%, which was similar to that in other studies; however, long-term follow-up and large-scale prospective studies are needed for making an accurate comparison.

The first limitation of this study is that it is difficult to use the tongue-in-groove technique if there is no rigid bone to support the implant or a defect to insert the implant. In particular, it has a disadvantage as it is difficult to use this method when the defect is not clear due to a trap door fracture in children. Second, it is difficult to use this method for reconstruction of the inferomedial orbital strut. Since it does not provide a strong fixation force, in this case, it is better to use a customized titanium plate to achieve solid fixation. Third, there is a limitation of the lack of long-term follow-up for more than several years. Long-term follow-up is required to check for additional side effects. It would be best to have a CT scan 3 months after surgery to see if there is a change in the position of the implant. Comparing only physical examination with clinical course is a major limitation of this study. Fourth, there is no holding force as strong as a screw. However, on the contrary, it is also convenient to remove the implant without a special operation; thus, it can be considered as an advantage that it can be easily removed if there is a problem with the implant. Fifth, there is a limitation of studies without a control group. Tongue-in-groove techniques were used if inclusion criteria were met, and usual surgical methods were used if they were not. Therefore, we were unable to establish an appropriate control group. Later, if the inclusion criteria are met, it is necessary to set up a control group with the usual surgical method. Lastly, a small retrospective single-center study needs to be followed by a multicenter prospective study in the future.

## 5. Conclusions

In conclusion, the tongue-in-groove technique has an advantage of fixing the implant to the defect site effectively, easily, and inexpensively. It is particularly short in terms of the molding time and is free to modify as needed. In addition, damage to the surrounding tissues can be minimized during the process of fixing the implant, and, if necessary, removing the implant is easy. Finally, it has the advantage of being easy to operate even in deep and narrow spaces. If this technique is used for appropriate surgical indications, it can be used as one of the options for fixing the implant.

## Figures and Tables

**Table 1:** Demographics.

		*n* = 138	
Sex	Male	115	(83.3%)
	Female	23	(16.7%)
Cause	Human trouble	46	(33.3%)
	Direct trauma	33	(23.9%)
	Slip down	39	(28.3%)
	Traffic accident	16	(11.6%)
	Fall	4	(2.9%)
Location of fracture	Inferior	55	(39.8%)
	Medial	55	(39.8%)
	Inferior + Medial	28	(20.4%)

**Table 2:** Comparison of Preoperative and Postoperative Orbital Volume Differences and Orbital Volume Reconstruction Rate between the Affected and Unaffected Eyes.

		Mean ± SD	*p*
**OV (mm^3^)**	Preoperative difference	2107.35 ± 1216.62	0.1129
	Postoperative difference	583.27 ± 891.21	<0.0001
**OVR (%)**	Preoperative OVR	87.15 ± 7.75	0.4421
	Postoperative OVR	94.21 ± 6.06	<0.0001

SD, standard deviation. OV, orbital volume. OVR, orbital volume reconstruction rate.

**Table 3:** Preoperative and postoperative complications.

	Preoperative	Postoperative
**Diplopia**	42 (30.4%)	22 (15.9%)
**Hypoesthesia**	19 (13.8%)	15 (10.9%)
**Enophthalmos**	12 (8.6%)	6 (4.3%)
**Ectropion**	0	1 (0.7%)
**Implant migration**	0	1 (0.7%)
**Retrobulbar hematoma**	1 (0.7%)	0 (0%)

## Data Availability

Data are contained within the article.
